# Haemorrhagic Pseudotumour Following Metal-on-Metal Hip Replacement

**DOI:** 10.7759/cureus.15541

**Published:** 2021-06-09

**Authors:** Joshua L Filer, James Berstock, Ynyr Hughes-Roberts, Julian Foote, Harvey Sandhu

**Affiliations:** 1 Trauma and Orthopaedics, Royal United Hospitals Bath, Bath, GBR; 2 Radiology, Royal United Hospitals Bath, Bath, GBR

**Keywords:** pseudotumour, haemorrhage, bleeding, hip arthroplasty, metal-on-metal

## Abstract

We present a unique report of a spontaneous haemorrhage into a pseudotumour five years following revision surgery for failed metal-on-metal hip arthroplasty. The patient sustained no trauma, was not taking anticoagulants and had no bleeding disorder. Rapid progression in the size of the pseudotumour caused significant symptoms and functional impairment. Surgical excision was recommended by a national specialist centre, but with conservative management, significant regression of the pseudotumour was noted, with complete resolution of symptoms. This case is the first report of haemorrhage into a pseudotumour, which is an important differential and can be managed non-operatively.

## Introduction

It has been widely documented that metal-on-metal hip arthroplasty is associated with adverse reactions to metal debris [1-5]. This reaction can lead to local tissue necrosis, fluid collection and the formation of inflammatory soft tissue masses known as ‘pseudotumours’ [2,3,5]. These pseudotumours are not uncommon although reported incidences vary with different implants, ranging from 0.6% to 61% [6-8].

Pseudotumours can be asymptomatic, but they can also cause pain, instability and gait disturbances, leading to the need for revision arthroplasty and/or excision of the soft tissue mass [3,9]. Although pseudotumours have also been implicated in lymphovascular complications resulting in venous thromboembolic events and swelling [10,11], to the best of our knowledge, spontaneous haemorrhage into a pseudotumour has not been described in the literature.

Although pseudotumours have been studied extensively [5,12-15], little is known about their natural history and controversy exists over their management, especially in asymptomatic patients [3,13,16]. Reports in the literature show that pseudotumours can grow in size, remain static, reduce in size and spontaneously resolve [13,16,17]. As a result of this varied natural history, it is difficult to decide how best to manage them both in the short and long term.

We describe a case of a pseudotumour that occurred at the site of a metal-on-metal hip replacement, which five years later had a large bleed into it with a late presentation. To our knowledge, this is the first case report of a haemorrhage into a pseudotumour. This is an important differential to recognise on cross-sectional imaging because it may not require surgical intervention.

## Case presentation

**Figure 1:**
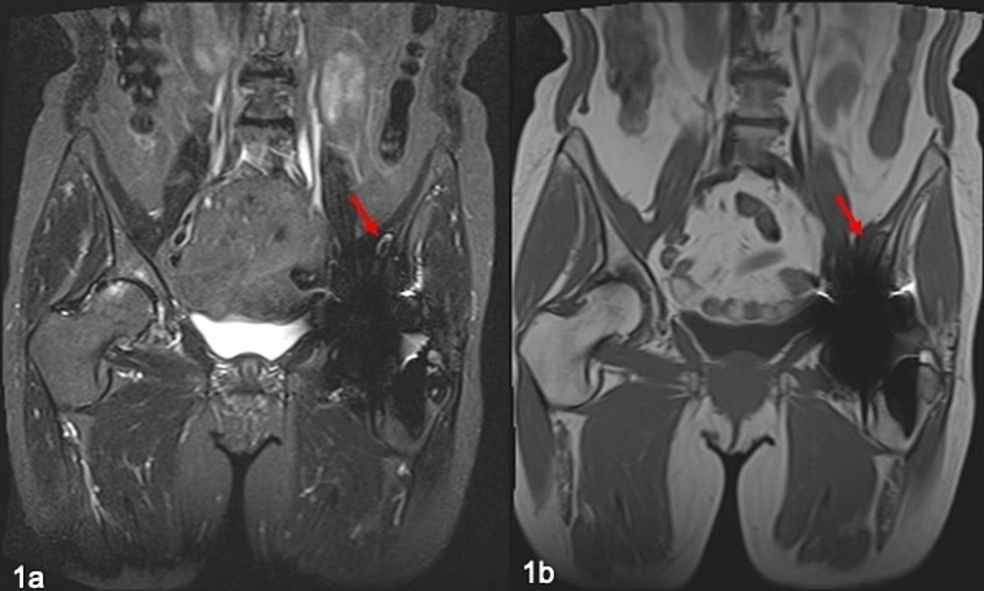
MRI of pseudotumour (red arrows) following metal-on-metal left hip arthroplasty in 2011. (a) Coronal STIR; (b) coronal T1. MRI: magnetic resonance imaging; STIR: short TI inversion recovery

A 62-year-old woman underwent a left metal-on-metal total hip replacement in 2008 (CORAIL®, PINNACLE with a 36 mm bearing). After three years, in 2011, this patient presented with significant ongoing symptoms. She reported that she had suffered from pain and a sensation of clicking and clunking since her initial surgery. On questioning, she felt it was never right and thought it felt too big. As a result, she was fully investigated. As part of the workup, metal artefact reduction sequence magnetic resonance imaging (MARS-MRI) showed there was a metal-on-metal reaction with a pseudotumour present within the pelvis (Figure 1). The mass, albeit small, was cystic in nature with a characteristic rim of low signal on both T1 and short TI inversion recovery sequences in keeping with haemosiderin deposition. Revision of the hip replacement was then performed in 2012 and the bearing was changed from a metal-on-metal bearing to a ceramic on highly cross-linked polyethylene bearing (trabecular metal component with the BioBall® system applied to the CORAIL® stem). Intra-operatively, there was a hip joint effusion, with markedly darkened abnormal intracapsular tissue. There was no other significant macroscopic pathology seen with no evidence of significant muscle or bone damage using the posterior approach. Following this revision procedure, the patient felt significantly better.

The patient returned to a high level of activity including gardening. However, five years later, the patient presented with spontaneous substantial bruising at the lateral aspect of the hip as well as bruising medially in the thigh. There was no history of trauma or injury. The pain was so severe that her mobility and ability to perform daily living activities were significantly impaired and morphine patches were required for a period of six months.

**Figure 2:**
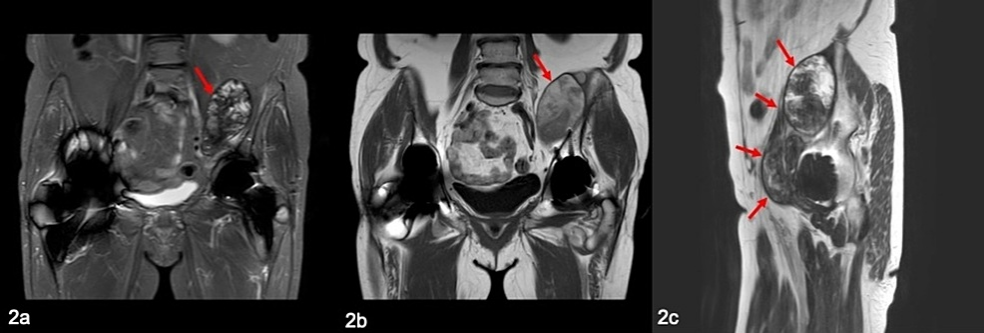
MRI of enlarging pseudotumour (red arrows) with evidence of haemorrhage in 2018. (a) Coronal STIR; (b) coronal T1; (c) sagittal T2. MRI: magnetic resonance imaging; STIR: short TI inversion recovery

The patient was investigated with MARS-MRI, which showed a substantial interval increase in the size of the residual pseudotumour. The persistence of the characteristic low signal rim confirmed the expansion of the mass. New, diffuse areas of high T1 signal were seen throughout the mass in keeping with areas of haemorrhage. The pseudotumour was retroperitoneal, extending into the femoral canal measuring 30 × 30 × 140 mm (Figure 2). Inflammatory markers and metal ion concentrations were reassuringly low. An aspiration of the hip was performed which confirmed blood products. Subsequent microscopy and culture investigations failed to detect any evidence of infection. A further MARS-MRI showed an increase in the size of this pseudotumour to 48 × 42 × 150 mm.

The patient was referred for a second opinion at a national unit renowned for its experience with metal-on-metal pathology. Haemorrhagic pseudotumours had not previously been identified at that unit either. After careful consideration, surgery was offered to the patient to excise this tumour in a combined approach with the orthopaedic and vascular teams. However, the patient was concerned with the invasiveness of the surgery that was required and declined.

The patient was closely followed up, and at her last review, her symptoms had improved. The improvement in symptoms has allowed the patient to return to a good level of function such as gardening, albeit with mild intermittent discomfort.

**Figure 3:**
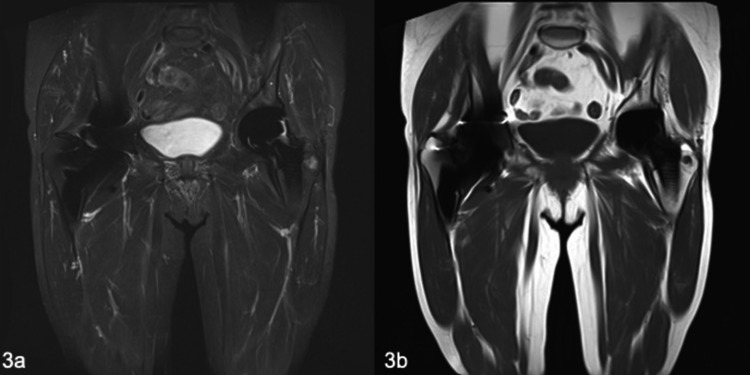
MRI of the pseudotumour in 2020 showing significant regression in size. (a) Coronal STIR; (b) coronal T1. MRI: magnetic resonance imaging; STIR: short TI inversion recovery

Follow-up MARS-MRI scans have been performed, most recently in 2020, and there has been a substantial regression in the size of the pseudotumour, reducing to a size of 20 × 18 × 63 mm, which correlates with the improvement in the clinical picture, with the pseudotumour now barely visible on MRI (Figure 3).

## Discussion

The purpose of this paper is to highlight an unusual case of haemorrhage into a pseudotumour, and how this was managed conservatively with activity modification and analgesia with good outcomes, despite operative treatment initially being considered.

Although it is recognised that pseudotumours can progress, regress, resolve or remain static [16,17], this case adds to our knowledge of the natural history and pathology of pseudotumours arising following metal-on-metal hip arthroplasty. In the case presented here, the pseudotumour was found to progress initially, causing significant pain and functional impairment. However, over a longer follow-up, it spontaneously regressed, with a commensurate improvement in symptoms and function.

## Conclusions

To the best of our knowledge, there are no other reported cases of bleeding into a pseudotumour associated with metal-on-metal hip arthroplasty. This case presented a significant clinical challenge in decision-making regarding the appropriate management. Accordingly, appropriate multidisciplinary team advice and input were sought from radiologists, vascular surgeons and a national specialist unit with good experience managing metal-on-metal arthroplasty-related complications. Although it was felt that surgical excision of the pseudotumour would be beneficial, the patient declined the proposed treatment and decided to be managed conservatively with good outcomes both clinically and radiographically.

This case shows that a haemorrhagic pseudotumour is an important differential of a growing pseudotumour, which can be successfully managed conservatively.
